# Psychoactive substance use and associated factors among Mohammed first university students, Oujda, Morocco: a cross-sectional study

**DOI:** 10.1186/s12889-024-19507-5

**Published:** 2024-07-23

**Authors:** Adil Essadi, Hanane Aissaoui, Asmae Yeznasni, Asmae Lekfif, Sanae Sebbar, Mariam Atassi, Naima Abda

**Affiliations:** Faculty of Medicine and Pharmacy, Mohammed First University, Oujda, Morocco

**Keywords:** Psychoactive substance use, University students, WHO ASSIST, Morocco, Alcohol use, Tobacco use, Drug use

## Abstract

**Background:**

The use of psychoactive substances, including tobacco, alcohol, and others, remains a major public health problem. However, few studies have been conducted on Moroccan university students. This study aimed to assess the prevalence and associated factors of substance use among students at Mohammed First University, Oujda, Morocco.

**Methods:**

We conducted this cross-sectional study with students enrolled at one of the Mohammed First University of Oujda’s institutes as part of the 2021–202 academic year. We used a self-administered survey to collect data anonymously. We cleaned the data and then entered it into IBM SPSS Statistics 21 for analysis. Data analysis involved descriptive statistics as well as univariate and multivariate analysis. We considered a *P* value < 0.05 as the level of significance.

**Results:**

In this study, out of 500 students we asked to complete the survey, 478 responded; the response rate was 95.6%. The average age was 21.1 ± 3.0, and the M/F sex ratio was 0.97. The lifetime prevalence of psychoactive substance use among Oujda University students was 28.7%. The most commonly used substances were tobacco (24.1%), alcohol (15.9%), cannabis (13.4%), sedatives (6.9%), stimulants (5.2%), and cocaine (4.4%). Male sex, age > 20 years, self-financing, school failure (one year repeated or more), the practice of a leisure activity, the presence of a personal medical or psychiatric history, and the presence of a family medical history were all significantly associated with the use of psychoactive substances.

**Conclusion:**

Our study revealed a significant prevalence of psychoactive substance use among university students in Oujda, highlighting the need for interventions at various levels. Further analytical studies are necessary to better understand the initiation and maintenance of psychoactive substance use and to identify all associated factors to enhance prevention strategies against substance use disorders.

**Supplementary Information:**

The online version contains supplementary material available at 10.1186/s12889-024-19507-5.

## Background

Throughout the course of human history, individuals have utilized a wide variety of plants, fungi, and other substances to modify their mood, sensations, emotions, thoughts, and cognitive capacities. Psychoactive substances possess the capacity to influence cognitive functions, mental processes, and consciousness. Psychoactive substances encompass a wide array of compounds, ranging from legally available and easily accessible substances such as caffeine, tobacco, and alcohol to controlled substances like psychotropic medications, in addition to illicit or prohibited substances such as cocaine [1–4].

The unregulated trade and use of psychoactive substances persisted for a considerable duration without significant medical or legal attention. Individuals would partake in the acquisition and consumption of these substances for cultural, religious, recreational, or therapeutic purposes without constraint. The 20th century marked a significant transformation in the realm of psychoactive substances, characterized by the emergence of medical definitions of substance use disorders [5] and the signing of international conventions aimed at regulating the import and export of drugs [6–8].

However, despite control measures, regulations, and restrictions aimed at containing the psychoactive substance market and limiting the use of illicit substances, both the number of users and the diversity of these substances persistently grow. The member states of the United Nations Office on Drugs and Crime (UNODC) reported the emergence of 1,124 new psychoactive substances in 2021 [9]. Moreover, according to the same office, a global estimate reveals that 284 million people between the ages of 15 and 64 used drugs at least once in 2021, accounting for 5.6% of the world population [10].

Unsurprisingly, cannabis stands out as the most widely used drug, with approximately 209 million people reported to have used it in 2021. Following closely are opioids, amphetamines, Ecstasy, and cocaine, with user numbers ranging from 21 to 61 million, as per the UNODC [11]. Regarding the use of legal psychoactive substances, especially tobacco and alcohol, the numbers become even more alarming. According to the World Health Organization (WHO), nearly 1.3 billion people aged 15 to 64 consumed tobacco in 2020, accounting for 22.3% of the global population [12]. In 2016, over 2.3 billion people aged 15 to 64 consumed alcohol at least once, representing 43% of the global population in that year [13]. Consequently, alcohol is the second most widely used psychoactive substance worldwide after caffeine [14].

In the global context of extensive production and consumption of legal and illicit psychoactive substances, our country is not an exception, as various figures and statistics indicate. A report published by the Economic, Social, and Environmental Council (CESE) revealed that in 2003, 4.1% of the Moroccan population aged 15 to 64 had engaged in drug use at least once, with cannabis emerging as the most prevalent drug at 3.9% [15]. Furthermore, a study conducted by the Ministry of Health in 2019, which examined common risk factors for noncommunicable diseases, reported that within the 30 days prior to the study, 1.7% of the adult Moroccan population had consumed alcohol, while 13.4% were active tobacco smokers [16].

While focusing on the use of psychoactive substances among Moroccan university students, a population estimated to exceed one million [17], which raises significant concerns for public health, it becomes evident that this area is severely underexplored within the national context. There is a lack of comprehensive literature regarding the prevalence of psychoactive substance use among this specific population, with only a limited number of localized or regional studies and surveys available. For instance, a notable survey conducted in 2005 among Cadi Ayyad University students in Marrakech revealed that 24.6% of the students used tobacco, 17.5% consumed alcohol, and 9.8% used cannabis [18]. Another survey conducted among students at Fes-Meknes universities revealed a lifetime prevalence of tobacco and alcohol use of 29.5% and 17.4%, respectively [19]. Through this study, our aim is to address this knowledge gap by investigating the prevalence, patterns, and factors associated with the use of psychoactive substances among students at Mohammed First University in Oujda.

## Materials and methods

We conducted this cross-sectional study in March 2022 to assess psychoactive substance use and associated factors among students enrolled at one of Mohammed First University of Oujda’s institutes as part of the 2021–2022 academic year.

The university is located in Oujda, in northeastern Morocco. It is one of the twelve public universities in Morocco and has 84 587 students (fall 2021) enrolled in ten academic institutions, including law and economics faculty, sciences faculty, medicine faculty and others [17]. The study population included all adult students enrolled at one of Mohammed First University of Oujda’s institutes as part of the 2021–2022 academic year. We excluded minors and students enrolled at professional or private institutes other than the university’s institutes.

To determine the minimum needed sample size, we used the OpenEpi 2013 website [20]. Considering a confidence interval of 95% and a margin error of 5%, and taking into account the total student population size of 84 587 students [17], along with an estimated prevalence of smoking of 16.2%, based on a 2016 study conducted among adolescents in Taza [21], which is in close proximity to Oujda, the sample size needed was 240, after considering a nonresponse rate of 15%.

We used a self-administered survey on paper to anonymously collect student variables (additional file 1), including sociodemographic variables (sex, age, nationality, marital status, etc.), academic variables (study field, level, self-reported academic performance, etc.), and substance use-related variables using ASSIST: the Alcohol, Smoking, and Substance Involvement Screening Test [22]. The ASSIST is an eight-item questionnaire developed by an international group of researchers and clinicians for the World Health Organization (WHO). In addition, the ASSIST also assigns a risk level for each substance use that falls between “lower”, “moderate” or “high.” The World Health Organization (WHO) recommends an appropriate level of intervention ranging from no intervention to brief intervention and more intensive treatment. Notably, the ASSIST score has demonstrated strong validity and test-retest reliability, further supporting its effectiveness as an assessment tool [22–26].

We employed two survey forms, one in French and the other in Arabic, to ensure local understanding and comprehensibility. We distributed the surveys on separate days in March 2022 across all institutes of the university, including classrooms, libraries, and cafeterias, using a convenience sampling method to ensure wide coverage and accessibility to a diverse student population. Upon collecting the surveys, we conducted a manual cleaning process, excluding questionnaires that did not meet the minimum criteria for completeness. Then we entered the cleaned data into IBM SPSS Statistics 21 for analysis.

Data analysis involved descriptive statistics as well as univariate and multivariate analysis. We reported quantitative data using means and standard deviation (SD), while qualitative data using numbers and percentages. We used the chi-square test (χ2) or Fisher’s exact test and student’s t-test to assess univariate association. Factors associated with lifetime psychoactive substance use on univariate analysis were entered in multivariate stepwise logistic regression model to investigate the risk factors of psychoactive substance use. We considered a *P* value < 0.05 as level the of significance.

## Results

Out of the 500 students who participated in the study, 478 completed the survey, resulting in a response rate of 95.6%. The study population had a sex ratio of 0.97, with 236 students (49.4%) being males and 242 (50.6%) being females. The age of the sample ranged from 18 to 54 years, with a mean age of 21.1 ± 3.0 years. Among the students, 241 (50.4%) were under the age of 20. Almost all of the participants were Moroccans (99.2%), except for four foreign students. More than half of the sample (56.6%) lived with their families. Table 1 summarizes the sociodemographic characteristics of the sample.

**Table 1:** Sociodemographic characteristics of Oujda University students *N* = 478

Characteristics	*n*	Valid percent
**Gender:**		
Female	242	50.6
Male	236	49.4
**Age**:		
≤ 20 years	241	50.4
> 20 years	237	49.6
**Nationality**:		
Moroccan	474	99.2
Non-Moroccan	4	0.8
**Origin region**:		
Oriental	386	80.8
Others	92	19.2
**Marital status**:		
Single	471	98.1
Married	7	1.5
Divorced	2	0.4
**Housing type**:		
Living alone	108	22.6
Living with family	270	56.5
Living in a shared apartment	99	20.7
**Parents viability**:		
Living parents	425	88.9
Deceased parent (s)	53	11.1
**Father’s level of education**:		
No education	91	19.1
Primary education	59	12.4
Secondary school	40	8.4
High school	73	15.3
University	214	44.9
**Mother’s level of education**:		
No education	165	34.5
Primary education	73	15.3
Secondary school	53	11.1
High school	75	15.7
University	112	23.4
**Financing type**:		
Family financing	399	86.2
Scholarship	234	50.3
Self-financing	69	14.7

Regarding academic characteristics, almost half of the respondents (49.8%) were in their third year or younger. Students from the law and economics faculty constituted 34.1% of the sample, while 23.4% were from the faculty of sciences, and 16.1% were medical students. The majority of students (77.2%) did not repeat a year in their academic career, while 15.0% repeated one year. Approximately 48.5% of the sample reported similar academic performance to their peers, while only 7.7% reported worse academic performance. Almost 57% of the sample engaged in leisure activities. Table 2 summarizes the academic characteristics of the sample.

**Table 2:** Academic characteristics of Oujda University students *N* = 478

Characteristics	*n*	Valid percent
**Study field:**		
Economics/Law	163	34.1
Mathematics/Natural Sciences	112	23.4
Medicine	77	16.1
Applied Sciences	45	9.4
Arts/Humanities	41	8.6
Commerce and Management	28	5.9
Others	12	2.5
**Study year**:		
1st year	115	24.9
2nd year	115	24.9
3rd year	161	34.8
4th year	35	7.6
5th year	20	4.3
6th year	9	1.9
7th year	7	1.5
**Academic repetition**:		
Yes	111	23.3
No	366	76.7
**Self-assessment of academic performance**:		
Much better	42	8.8
Better	167	34.9
Same	232	48.5
Worse	30	6.3
Much worse	7	1.5
**Engagement in leisure activities**:		
Yes	270	43.4
No	207	56.6

In terms of health characteristics, the majority of the sample (94.6%) reported being in good health, 5.4% reported having a chronic disease, and nearly 3.0% reported having a psychiatric disorder. Additionally, almost a quarter of the participants reported a family history of chronic disease, and 3.6% reported a family history of a psychiatric disorder. Table 3 summarizes the health characteristics of the sample.

**Table 3:** Health characteristics of Oujda University students *N* = 478

Characteristics	*n*	Valid percent
**Personal medical history:**		
Yes	26	5.4
No	452	94.6
**Personal psychiatric history**:		
Yes	14	2.9
No	464	97.1
**Family medical history**:		
Yes	110	23.0
No	368	77.0
**Family psychiatric history**:		
Yes	17	3.6
No	461	96.4

The lifetime prevalence of psychoactive substance use among Oujda University’s students was 28.7%. Specifically, 24.1% reported using tobacco, 15.9% used alcohol, and 13.4% used cannabis. In the past three months, the prevalence of tobacco, alcohol, and cannabis use were 17.2%, 11.5%, and 9.0%, respectively. Table 4 summarizes the characteristics of psychoactive substance use. When assessing each student’s involvement score based on the ASSIST score, we found that 47.7%, 25.2%, and 22.4% of tobacco, cannabis, and alcohol users, respectively, required “brief intervention”. Only 10.3% of tobacco users and 5.6% of cannabis users required “more intensive treatment”, while the majority of users of other substances did not require any intervention. Table 5 summarizes the ASSIST-recommended interventions by substance for Oujda university students.

**Table 4:** Psychoactive substance use characteristics of Oujda University students

substances	Lifetime use (*n* = 137)		Past three months use (*n* = 137)	
	Yes *N* (Valid percent)	No *N* (Valid percent)	Yes *N* (Valid percent)	No *N* (Valid percent)
Tobacco	115 (84.6)	21 (15.4)	82 (62.1)	50 (37.9)
Alcohol	76 (55.9)	60 (44.1)	55 (41.7)	77 (58.3)
Cannabis	64 (47.4)	71 (52.6)	43 (32.6)	89 (67.4)
Cocaine	21 (15.7)	113 (84.3)	10 (7.6)	122 (92.4)
Amphetamine-type stimulants	25 (18.4)	111 (81.6)	11 (8.3)	121 (91.7)
Sedatives	33 (24.3)	103 (75.7)	22 (6.7)	110 (83.3)
Opioids	14 (10.3)	122 (89.7)	7 (5.3)	125 (94.7)
inhalants	20 (14.7)	116 (85.3)	8 (6.1)	124 (93.9)
Hallucinogens	11 (8.1)	125 (91.9)	5 (3.8)	127 (96.2)
Others	6 (4.4)	130 (95.6)	1 (0.8)	131 (99.2)

**Table 5:** ASSIST-recommended interventions by substance for Oujda university students

substances	No intervention *n* (valid percent)	Brief intervention *n* (valid percent)	More intensive treatment *n* (valid percent)
Tobacco	44 (41.5)	51 (48.1)	11 (10.4)
Alcohol	82 (76.6)	24 (22.4)	1 (0.9)
Cannabis	74 (69.2)	27 (25.2)	6 (5.6)
Cocaine	99 (92.5)	7 (6.5)	1 (0.9)
Amphetamine-type stimulants	99 (92.5)	7 (6.5)	1 (0.9)
Sedatives	92 (86.8)	12 (11.3)	2 (1.9)
Opioids	102 (96.2)	4 (3.8)	0 (0.0)
inhalants	103 (96.3)	4 (3.7)	0 (0.0)
Hallucinogens	104 (97.2)	3 (2.8)	0 (0.0)
Others	0 (0.0)	0 (0.0)	0 (0.0)

We examined the association between psychoactive substance use and the sociodemographic, academic, and health characteristics of the sample. We found that males were more likely to use substances than females (*p* < 0.001), with 47.0% of male students using substances compared to only 10.7% of female students. The likelihood of substance use was significantly higher among students above 20 years of age than among those between 18 and 20 years old (*p* < 0.001). Students who had their own source of income were more likely to use substances (*p* < 0.001).

Furthermore, students who repeated at least one year in their academic career were more likely to use substances than those who did not repeat (*p* < 0.001). Engaging in a leisure activity was significantly associated with a higher likelihood of psychoactive substance use compared to those who did not participate in such activities (*p* < 0.001).

Moreover, students who reported having a chronic disease, a psychiatric disorder, or a family history of chronic disease were more likely to use substances than those without these factors (*p* < 0.005). Table 6 summarizes the sociodemographic, academic, and health characteristics associations with psychoactive substance use among the study participants.

**Table 6:** Sociodemographic, academic, and health characteristics associated with psychoactive substance use among Oujda University students

Lifetime use of psychoactive substances			*n* = 137
	Yes *n* (%)	No *n* (%)	*P*_value_
**Gender:**			
Female	26 (10.7)	216 (89.3)	< 0.0001
Male	111 (47.0)	125 (53.0)	
**Age**:			
≤ 20 years	49 (20.3)	192 (79.7)	< 0.0001
> 20 years	88 (37.1)	149 (62.9)	
**Nationality**:			
Moroccan	136 (28.7)	338 (71.3)	0.675
Non-Moroccan	1 (25.0)	3 (75.0)	
**Origin region**:			
Oriental region	109 (27.1)	293 (72.9)	0.073
Other regions	27 (37.5)	45 (62.5)	
**Marital status**:			
Married	3 (42.9)	4 (57.1)	0.322
Single or divorced	134 (28.5)	337 (71.5)	
**Housing type**:			
Living alone	35 (32.4)	73 (67.6)	0.498
Living with family	72 (26.7)	198 (73.3)	
Living in a shared apartment	30 (30.3)	69 (69.7)	
**Viability of parents**:			
Living parents	120 (28.2)	305 (71.8)	0.560
Deceased parent(s)	17 (32.1)	36 (67.9)	
**Father’s level of education**:			
No/primary education	37 (24.7)	113 (75.3)	0.208
Secondary/high school/university	99 (30.3)	228 (69.7)	
**Mother’s level of education**:			
No/primary education	62 (26.1)	176 (73.9)	0.209
Secondary/high school/university	75 (31.3)	165 (68.8)	
**Scholarship recipient**:			
Yes	59 (25.2)	175 (74.8)	0.100
No	76 (32.1)	161 (67.9)	
**Family financing**:			
Yes	110 (27.6)	289 (72.4)	0.104
No	24 (37.5)	40 (62.5)	
**Self-financing**:			
Yes	39 (56.5)	30 (43.5)	< 0.0001
No	96 (24.0)	304 (76.0)	
**Study field**:			
Medicine	28 (36.4)	49 (63.6)	0.48
Commerce and Management	9 (32.1)	19 (67.9)	
Economics/Law	47 (28.8)	116 (71.2)	
Mathematics/Natural Sciences	30 (26.8)	82 (73.2)	
Applied Sciences/Other	15 (26.3)	42 (73.7)	
Arts/Humanities	8 (19.5)	33 (80.5)	
**Study cycle**:			
First cycle	47 (20.4)	183 (79.6)	< 0.0001
Second/third cycle	84 (36.2)	148 (63.8)	
**Academic repetition**:			
Yes	51 (45.9)	60 (54.1)	< 0.0001
No	85 (23.2)	281 (76.8)	
**Self-assessment of academic performance**:			
Higher/Similar	123 (27.9)	318 (72.1)	0.199
Lower	14 (37.8)	23 (62.2)	
**Engagement in leisure activities**:			
Yes	81 (39.1)	126 (60.9)	< 0.0001
No	55 (20.4)	215 (79.6)	
**Personal medical history**:			
Yes	12 (46.2)	14 (53.8)	0.043
No	125 (27.7)	326 (72.3)	
**Personal psychiatric history**:			
Yes	9 (64.3)	5 (35.7)	0.005
No	126 (27.4)	334 (72.6)	
**Family medical history**:			
Yes	40 (36.4)	70 (63.6)	0.045
No	97 (26.5)	269 (73.5)	
**Family psychiatric history**:			
Yes	5 (29.4)	12 (70.6)	0.565
No	131 (28.6)	327 (71.4)	

After conducting a multivariate logistic regression analysis, we found that male students had a considerably higher likelihood of using psychoactive substances than female students (OR: 7.56, 95% CI: [7.38–13.04]). Students with a psychiatric history had a significantly higher likelihood of substance use than those without (OR: 12.91, 95% CI: [3.29–5.55]). Self-financed students had a higher risk of using psychoactive substances than non-self-financed students (OR: 2.62, 95% CI: [1.43–4.81]). Furthermore, students in the second or third cycle were more likely to use substances than first-cycle students (OR: 2.16, 95% CI: [1.34–3.49]) (Table 7).

**Table 7:** Multivariate logistic regression analysis showing characteristics associated with lifetime psychoactive substance use among Oujda University students

	OR/ [95%CI]	*P*_value_
**Gender:**		
Female	1	< 0.001
Male	7.56 [7.38–13.04]	
**Self-financing**:		
No	1	0.002
Yes	2.62 [1.43–4.81]	
**Study cycle**:		
First cycle	1	0.001
Second/third cycle	2.16 [1.34–3.49]	
**Personal psychiatric history**:		
Yes	1	< 0.001
No	12.91 [3.29–5.55]	

## Discussion

This study aims to evaluate the lifetime and past three-months prevalence of psychoactive substance use among students at Oujda University while also exploring its associations with sociodemographic, academic, and health factors. We employed the Alcohol, Smoking, and Substance Involvement Screening Test (ASSIST) to assess the lifetime and past three-months use of various substances, including alcohol, tobacco, cannabis, cocaine, amphetamine-type stimulants, sedatives, opioids, inhalants, hallucinogens, and “other” substances [22], and also to evaluate the level of ASSIST-recommended interventions for each substance [22–26].

The study found that among Mohammed First University students, the overall lifetime and past three-months prevalence of psychoactive substance use were 28.7% and 27.6%, respectively. Unfortunately, there is a lack of comparative data from other universities in the kingdom to contextualize these results. In comparison to other studies conducted worldwide, our findings revealed significantly lower rates of psychoactive substance use. For instance, a cross-sectional study of 763 undergraduate students in Nigeria reported a high lifetime rate of 84.5% [27], while a systematic review and meta-analysis conducted in Ethiopia found an overall prevalence of 32.28% among students [28]. Similarly, a study conducted in France between 2015 and 2017 reported a lifetime use rate of 83.32% for at least one psychoactive substance [29]. These prevalence rates observed in different countries are notably higher than those found in our study and in other Muslim-majority countries such as Turkey [30], where the estimated prevalence is 6.4%. This discrepancy may suggest the influence of religion on the variations in lifetime substance use prevalence across different regions [31, 32].

Notably, our research revealed tobacco as the most commonly used psychoactive substance, with a lifetime prevalence of 24.1% and a past three-months prevalence of 17.2%. These findings were consistent with similar studies conducted at other universities. For instance, at Marrakech University [18], 24.6% of students reported tobacco consumption during their lifetime, while at Fes and Meknes Universities [19], the figure stood at 29.5%. Comparatively, our observed lifetime prevalence exceeded the rates of tobacco use in Ethiopia [33], with a prevalence of 7.4%, and Nigeria [27], with a prevalence of 13.6%. Conversely, it fell below the prevalence rates observed in Lebanon [34] at 36.3%, France [29] at 30.6%, and Spain [35] at 78.8%.

Alcohol emerged as the second most commonly used substance, with a lifetime prevalence of 15.9% and a past three-months prevalence of 11.5%. These findings align with similar studies conducted at other universities, such as Marrakech University [18], where 17.5% of students reported alcohol consumption during their lifetime, Fes and Meknes University [19], where the prevalence stood at 17.4%. However, when comparing our observed lifetime prevalence, it was lower than the rates of alcohol use in all the aforementioned studies. For instance, in Ethiopia [33], the prevalence was 16.0%, while in Nigeria [27], it was significantly higher at 82.7%. Similarly, Spain [35] reported a prevalence of 98.4%, France [29] reported 73.7%, and Lebanon [34] reported 47.7%.

With a lifetime prevalence of 13.4% and a past-three-months prevalence of 9.0%, cannabis emerged as the third most commonly used substance. These findings fall within the range of comparable studies conducted at other universities, such as Marrakech University [18], where 9.8% of students reported lifetime cannabis consumption, and Fes and Meknes Universities [19], where the prevalence was 16.1%. However, when comparing the prevalence of cannabis use in other countries, a similar pattern emerges, as seen with tobacco use. Sub-Saharan countries tend to have lower prevalences, while European countries have higher prevalences. For instance, in Ethiopia [33] the prevalence was 4.5%, while in Nigeria [27] it was 7.6%. In contrast, Spain [35] reported a prevalence of 17.7%, and France [29] reported a significantly higher prevalence of 57%. We can attribute this similarity to the fact that students often smoke cannabis by combining tobacco and cannabis in the form of joints or blunts.

Our research findings revealed that 6.9% of students reported having used sedatives and sleeping pills at some point in their lives, with 4.6% indicating recent usage within the past three months. It is noteworthy that our study’s results closely align with those reported by Fes and Meknes universities [19], where the lifetime usage rate was found to be 5.1%. This similarity suggests a consistent pattern within the national context. However, when compared to broader trends observed in other regions, our study indicates comparatively lower prevalence rates. For example, in Lebanon [34], the prevalence of sedative and sleeping pill usage was recorded at 11.0%, while in Nigeria [27], it reached a significantly higher rate of 14.2%. Most concerning is the prevalence in Spain [35], which stood at an alarming 18.8%.

Turning our attention to cocaine use, we found lifetime and past-three-months prevalences of 4.4% and 2.1%, respectively, among our sample. Despite these relatively lower figures, it is still a cause for concern given the substantial evidence of its short- and long-term health consequences [23, 36]. Comparatively, the prevalence of cocaine use among university students was lower in sub-Saharan countries, with Nigeria [27] reporting 1.4% and Ethiopia [33] reporting 2.5%. In contrast, France [29] exhibited a prevalence of 7.0%, while Spain [35] reported a significantly higher prevalence of 19.1%.

Multiple factors could contribute to the variations in the lifetime prevalence of substance use across different countries and regions. These factors may include socioeconomic status, cultural differences, religion, the availability and accessibility of psychoactive substances, and the level of awareness and education on substance use. Additionally, it is important to acknowledge that sample characteristics could play a role in these variations.

Our findings revealed that a mere 1.3% of students opted for the “other” category when reporting their substance use, suggesting that the ASSIST [22] tool is generally adequate for evaluating psychoactive substance use. Nevertheless, the inclusion of supplementary categories and a wider range of substances, considering regional variations, could enhance its effectiveness in assessing psychoactive substance use among diverse populations.

Based on the results obtained from ASSIST, our findings indicate that the majority of students who use substances do not require any intervention. However, it is important to note that 10.3% of tobacco users, 5.6% of cannabis users, and 1.9% of students using sedative pills would benefit from “more intensive treatment” as recommended by the World Health Organization (WHO) [22]. These individuals necessitate a comprehensive clinical assessment and specialized treatment tailored to address their specific substance use concerns. Moreover, our study reveals that a significant proportion of students would benefit from “brief intervention.” Among tobacco users, 47.7% would benefit from this approach, along with 25.2% of cannabis users, 22.4% of alcohol users, and 11.2% of sedative pill users. Brief interventions involve providing feedback through the ASSIST feedback report card, distributing the informative “Self-help strategies for cutting down or stopping substance use: a guide” booklet [37], and offering specific substance-related information to assist them in reducing or ceasing their substance use [22].

After conducting the univariate analysis, it became evident that there was a significant correlation between the consumption of psychoactive substances and male gender (*P* < 0.0001), as well as age over 20 years (*P* < 0.0001). These associations have been consistently reported in previous studies [18, 19, 30, 33, 34], suggesting a robust pattern. The reasons behind these associations can be attributed to diverse social and cultural factors that tend to normalize substance consumption among men more than women and among young adults more than adolescents.

The observed significant association (*P* = 0.005) between substance consumption and anxiety and/or depressive disorders is a two-way relationship. It is important to note that internalized disorders, such as depressive and anxiety disorders can both contribute to and result from substance consumption or substance use disorder [38]. The study by Christie et al. [39] provides further evidence, showing that a preexisting history of depressive or anxiety disorders increases the risk of developing a substance use disorder in the future. These findings are consistent with other studies in the literature [40–42].

Moreover, we discovered a noteworthy and statistically significant association (*P* < 0.0001) between the consumption of psychoactive substances and participation in leisure activities. This association can be explained through a biosocial perspective on personality [43, 44]. According to Cloninger [43, 44], novelty seeking represents one of the fundamental traits of personality and refers to an individual’s tendency to seek out new and exciting experiences, sensations, and stimulation, including participating in leisure activities. Several authors [44–46] have found a significant link between novelty seeking and the use of alcohol, tobacco, and other substances.

In addition to our findings, we discovered a significant association (*P* < 0.0001) that is seldom reported, namely the link between substance use and academic failure, assessed by the number of repeated school years. Interestingly, the study conducted in Marrakech [18] reported a similar result. This association implies the potential involvement of psychosocial factors and personality traits in both academic failure and substance use.

Furthermore, we identified a significant association (*P* < 0.0001) between substance use and self-financing, indicating that students who have the ability to generate income are more likely to engage in substance consumption than their counterparts. Interestingly, the study in Fes and Meknes universities [18] reported a similar association, where students who received pocket money exceeding 150 euros were more susceptible to use substances. These findings suggest a potential relationship between financial resources and substance use among students.

By conducting this study, we achieved our predefined objectives and effectively utilized the data collected during the survey. However, it is important to acknowledge several limitations that deserve emphasis. First, our study relied on a cross-sectional survey design, which poses challenges in establishing causal inferences. Furthermore, the limited sample size, the sampling procedure and difficulties in accessing university students, given their diverse schedules and absenteeism, raise concerns about the representativeness of our findings and could be subject to selection bias. Another limitation is the reliance on self-assessed, subjective data, instead of clinical (assessments by healthcare professionals) and biological (blood tests, urine tests) evaluations of substance use, which can be prone to biases and inaccuracies, including recall bias. Given the sensitive nature of the subject matter from both sociocultural and judicial perspectives, the reported values may not necessarily reflect the true reality due to social desirability bias.

While our study successfully met its objectives and utilized the available data, it is essential to recognize these limitations. Future research should consider alternative study designs with larger and more diverse samples to better assess the prevalence of substance use and identify its associated risk factors. These efforts will contribute to the enhancement and guidance of national initiatives focused on reducing substance use and preventing related disorders.

## Conclusion

Our study revealed a significant prevalence of psychoactive substance use among university students in Oujda, particularly in terms of tobacco and alcohol consumption, highlighting the need for interventions at various levels. Further analytical studies are necessary to better understand the initiation and maintenance of psychoactive substance use and to identify all associated factors, to enhance prevention strategies against substance use disorders.

### Electronic supplementary material

Below is the link to the electronic supplementary material.

Supplementary Material 1

## Data Availability

The datasets used and/or analyzed during the current study are available from the corresponding author on reasonable request.
