# Supplementary material for: Psychoactive substance use and associated factors among Mohammed first university students, Oujda, Morocco: a cross-sectional study
Source: BMC Public Health. 2024 Jul 23;24:1961. doi: 10.1186/s12889-024-19507-5 (PMC11264429; doi:10.1186/s12889-024-19507-5)
Supplement: Supplementary file 1 — Supplementary Material 1 [file 12889_2024_19507_MOESM1_ESM.pdf]

Royaume du Maroc
Université Mohammed Premier
Oujda

Read this first please

This questionnaire aims to "Estimate the prevalence of psychoactive substance use among university students in Oujda" as part of a study organized by the laboratory of epidemiology, clinical research, and community health. We kindly invite you to fill out this questionnaire to help us conduct this study effectively.

This questionnaire consists of multiple-choice questions. If you do not find the ideal answer among the choices provided, please select the one that you find closest to reality.

We would like to inform you that by participating in this study:

- **You acknowledge that your participation is entirely voluntary.**
- **Your responses will be used solely for scientific purposes** (conferences, articles, scientific journals, and other studies).
- **Your responses will be treated anonymously and confidentially.**

Thank you for the time you will dedicate to completing this questionnaire.

A. <u>Sociodemographic Data:</u>

1. Gender: Female ☐ Male ☐
2. Age in years: ………… years
3. Nationality: ………………………………….
4. City of origin: ……………………………
5. Marital status: Married ☐ Single ☐ Divorced ☐
6. Do you live: Alone ☐ With family ☐ In shared accomodation ☐
7. Are your parents alive: Yes ☐ No ☐
   If no, which one is deceased : (father / mother / both) : ……………………
8. What is the highest level of education your father has completed?
   None ☐ Primary ☐ Secondary ☐ High school ☐ University ☐
9. What is the highest level of education your mother has completed?
   None ☐ Primary ☐ Secondary ☐ High school ☐ University ☐
10. Financial source:
   - Scholarship : Yes ☐ No ☐
   - Parents : Yes ☐ No ☐
   - Self-financing : Yes ☐ No ☐
   If yes, pecify your income-generating activity : ……………………………………..

B. <u>University Study Data:</u>

1. In which field are you enrolled?
   Économics / Law ☐ Letters / Humanities ☐ Medicine ☐ Math / physics / Biology ☐ Commerce and management ☐ Applied sciences / Technology ☐ Others ☐
2. Year of study: ……………………………………
3. Have you ever repeated a year: Yes ☐ No ☐
   If yes, how many times: ………………………
4. Compared to your colleagues, how do you judge your academic performance?
   More performant ☐ Performant ☐ Similar ☐ Worse ☐ Much worse ☐
5. Do you engage in any leisure activity: Yes ☐ No ☐
   If yes, which one? …………………………………………………………

C. **Personal and Family Medical History:**

1. Are you being treated for a chronic disease? Yes ☐ No ☐.
   If yes, which one: …………………………………………………………...
2. Are you being treated for a psychiatric disorder? Yes ☐ No ☐.
   If yes, which one: …………………………………………………………...
3. Do you have a parent/sibling being treated for a chronic disease? Yes ☐ No ☐.
   If yes, which one: …………………………………………………………...
4. Do you have a parent/sibling being treated for a psychiatric disorder? Yes ☐ No ☐.
   If yes, which one: …………………………………………………………...

The following questions pertain to your consumption of alcohol, tobacco, and other drugs throughout your life and in the last 3 months, regardless of the method of consumption (smoked, ingested, sniffed, inhaled, injected, taken as a pill, etc.).

Please be assured that **all information collected will be treated in a strictly confidential manner**, including those concerning illicit or prohibited drugs.

Question 1:

In your life, which of the following substances have you ever used (non-medical use only)?	No	Yes
a. Tobacco products (cigarettes, chewing tobacco, cigars, etc.)		
b. Alcoholic beverages (beer, wine, spirits, etc.)		
c. Cannabis (marijuana, pot, grass, hash, etc.)		
d. Cocaine (coke, crack, etc.)		
e. Amphetamine-type stimulants (speed, meth, ecstasy, etc.)		
f. Inhalants (nitrous, glue, petrol, paint thinner, etc.)		
g. Sedatives or sleeping pills (Valium, Seresta, Stilnox, Rivotril, Alpraz, etc.)		
h. Hallucinogens (LSD, acid, mushrooms, trips, ketamine, etc.)		
i. Opioids (heroin, morphine, methadone, buprenorphine, codeine, etc.)		
j. Other – specify:		

-If your answer is "no" for all items, thank you for your participation, the questionnaire ends here.

-If you have consumed any substance at least once in your life, proceed to question 2.

Question 2:

During *the past three months,* how often have you used the substances you mentioned (first drug, second drug, etc)?	Never	Once or twice	Monthly	Weekly	Daily or almost daily
a. Tobacco products (cigarettes, chewing tobacco, cigars, etc.)					
b. Alcoholic beverages (beer, wine, spirits, etc.)					
c. Cannabis (marijuana, pot, grass, hash, etc.)					
d. Cocaine (coke, crack, etc.)					
e. Amphetamine-type stimulants (speed, meth, ecstasy, etc.)					
f. Inhalants (nitrous, glue, petrol, paint thinner, etc.)					
g. Sedatives or sleeping pills (Valium, Seresta, Stilnox, Rivotril, Alpraz, etc.)					
h. Hallucinogens (LSD, acid, mushrooms, trips, ketamine, etc.)					
i. Opioids (heroin, morphine, methadone, buprenorphine, codeine, etc.)					
j. Other – specify:					

-If you have not consumed any substance in the last 3 months, please skip to question 6.

-If you have consumed at least one substance in the last 3 months, continue with questions 3, 4, and 5.

Question 3:

During *the past three months,* how often have you had a strong desire or urge to use (first drug, second drug, etc)?	Never	Once or twice	Monthly	Weekly	Daily or almost daily
a. Tobacco products (cigarettes, chewing tobacco, cigars, etc.)					
b. Alcoholic beverages (beer, wine, spirits, etc.)					
c. Cannabis (marijuana, pot, grass, hash, etc.)					
d. Cocaine (coke, crack, etc.)					
e. Amphetamine-type stimulants (speed, meth, ecstasy, etc.)					
f. Inhalants (nitrous, glue, petrol, paint thinner, etc.)					
g. Sedatives or sleeping pills (Valium, Seresta, Stilnox, Rivotril, Alpraz, etc.)					
h. Hallucinogens (LSD, acid, mushrooms, trips, ketamine, etc.)					
i. Opioids (heroin, morphine, methadone, buprenorphine, codeine, etc.)					
j. Other – specify:					

Question 4:

During *the past three months*, how often has your use of (first drug, second drug, etc) led to health, social, legal or financial problems?	Never	Once or twice	Monthly	Weekly	Daily or almost daily
a. Tobacco products (cigarettes, chewing tobacco, cigars, etc.)					
b. Alcoholic beverages (beer, wine, spirits, etc.)					
c. Cannabis (marijuana, pot, grass, hash, etc.)					
d. Cocaine (coke, crack, etc.)					
e. Amphetamine-type stimulants (speed, meth, ecstasy, etc.)					
f. Inhalants (nitrous, glue, petrol, paint thinner, etc.)					
g. Sedatives or sleeping pills (Valium, Seresta, Stilnox, Rivotril, Alpraz, etc.)					
h. Hallucinogens (LSD, acid, mushrooms, trips, ketamine, etc.)					
i. Opioids (heroin, morphine, methadone, buprenorphine, codeine, etc.)					
j. Other – specify:					

Question 5:

During *the past three months*, how often have you failed to do what was normally expected of you because of your use of (first drug, second drug, etc)?	Never	Once or twice	Monthly	Weekly	Daily or almost daily
a. Tobacco products (cigarettes, chewing tobacco, cigars, etc.)					
b. Alcoholic beverages (beer, wine, spirits, etc.)					
c. Cannabis (marijuana, pot, grass, hash, etc.)					
d. Cocaine (coke, crack, etc.)					
e. Amphetamine-type stimulants (speed, meth, ecstasy, etc.)					
f. Inhalants (nitrous, glue, petrol, paint thinner, etc.)					
g. Sedatives or sleeping pills (Valium, Seresta, Stilnox, Rivotril, Alpraz, etc.)					
h. Hallucinogens (LSD, acid, mushrooms, trips, ketamine, etc.)					
i. Opioids (heroin, morphine, methadone, buprenorphine, codeine, etc.)					
j. Other – specify:					

Question 6:

Has a friend or relative or anyone else ever expressed concern about your use of (first drug, second drug, etc)?	No, never	Yes, in the past 3 months	Yes, but not in the past 3 months
a. Tobacco products (cigarettes, chewing tobacco, cigars, etc.)			
b. Alcoholic beverages (beer, wine, spirits, etc.)			
c. Cannabis (marijuana, pot, grass, hash, etc.)			
d. Cocaine (coke, crack, etc.)			
e. Amphetamine-type stimulants (speed, meth, ecstasy, etc.)			
f. Inhalants (nitrous, glue, petrol, paint thinner, etc.)			
g. Sedatives or sleeping pills (Valium, Seresta, Stilnox, Rivotril, Alpraz, etc.)			
h. Hallucinogens (LSD, acid, mushrooms, trips, ketamine, etc.)			
i. Opioids (heroin, morphine, methadone, buprenorphine, codeine, etc.)			
j. Other – specify:			

Question 7:

Have you ever tried to cut down on using (first drug, second drug, etc) but failed?	No, never	Yes, in the past 3 months	Yes, but not in the past 3 months
a. Tobacco products (cigarettes, chewing tobacco, cigars, etc.)			
b. Alcoholic beverages (beer, wine, spirits, etc.)			
c. Cannabis (marijuana, pot, grass, hash, etc.)			
d. Cocaine (coke, crack, etc.)			
e. Amphetamine-type stimulants (speed, meth, ecstasy, etc.)			
f. Inhalants (nitrous, glue, petrol, paint thinner, etc.)			
g. Sedatives or sleeping pills (Valium, Seresta, Stilnox, Rivotril, Alpraz, etc.)			
h. Hallucinogens (LSD, acid, mushrooms, trips, ketamine, etc.)			
i. Opioids (heroin, morphine, methadone, buprenorphine, codeine, etc.)			
j. Other – specify:			

Question 8:

	No, Never	Yes, in the past 3 months	Yes, but not in the past 3 months
Have you ever used any drug by injection (non-medical use only)?			

Thank you very much for your contribution.
